# SNP Set Association Analysis for Genome-Wide Association Studies

**DOI:** 10.1371/journal.pone.0062495

**Published:** 2013-05-03

**Authors:** Min Cai, Hui Dai, Yongyong Qiu, Yang Zhao, Ruyang Zhang, Minjie Chu, Juncheng Dai, Zhibin Hu, Hongbing Shen, Feng Chen

**Affiliations:** 1 Department of Epidemiology and Biostatistics, School of Public Health, Nanjing Medical University, Nanjing, China; 2 Section of Clinical Epidemiology, Jiangsu Key Laboratory of Cancer Biomarkers, Prevention and Treatment, Cancer Center, Nanjing Medical University, Nanjing, China; 3 State Key Laboratory of Reproductive Medicine, Nanjing Medical University, Nanjing, China; The University of Chicago, United States of America

## Abstract

Genome-wide association study (GWAS) is a promising approach for identifying common genetic variants of the diseases on the basis of millions of single nucleotide polymorphisms (SNPs). In order to avoid low power caused by overmuch correction for multiple comparisons in single locus association study, some methods have been proposed by grouping SNPs together into a SNP set based on genomic features, then testing the joint effect of the SNP set. We compare the performances of principal component analysis (PCA), supervised principal component analysis (SPCA), kernel principal component analysis (KPCA), and sliced inverse regression (SIR). Simulated SNP sets are generated under scenarios of 0, 1 and ≥2 causal SNPs model. Our simulation results show that all of these methods can control the type I error at the nominal significance level. SPCA is always more powerful than the other methods at different settings of linkage disequilibrium structures and minor allele frequency of the simulated datasets. We also apply these four methods to a real GWAS of non-small cell lung cancer (NSCLC) in Han Chinese population

## Introduction

It is widely believed that genetic variants play an important role in the etiology of common diseases risk or quantitative traits. In the last few years, we have witnessed the development of GWAS which have become a popular approach for identifying related genetic variation of complex diseases [1], [2]. Nowadays, with the rapid development in high throughput genotyping technology, millions of SNPs can be genotyped simultaneously from more than one thousand cases and controls in GWAS. Single-locus association tests (SLAT) is usually run to identify causal or associated SNPs of diseases. Such a SNP-by-SNP association study requests a multiple testing adjustment procedure to ensure overall appropriate type I error rate, such as Bonferroni correction and false discovery rates. As an example, each SNP should be tested at the level of 5e-8 to maintain the overall significance level at 0.05 for a GWAS including one million SNPs [3], which may be too stringent. Recently studies suggest that complex diseases are often caused by weak effect SNPs (relative risk *RR*< = 1.5), which results in poor statistical power after multiple correction [4], [5].

One way to deal with these challenges is to consider higher units for the analysis. Several studies have revealed that treat higher units instead of each genotyped SNP may alleviate the problems of intensive computation and multiple testing [6], [7], lead to more stable results and higher interpretability [8], [9]. It is possible that multiple loci association studies (MLAS) have higher power than testing each SNP individually [10]. Several methods have been proposed based on grouping SNPs into SNP sets as higher units. Gauderman et al. proposed a principal component based approach (PCA), by which several principal components (PCs) were extracted from the SNP set and regressed with the phenotype [11]. Gao et al. studied that KPCA-LRT was an effective and powerful gene-or region-based method for GWAS datasets, especially under lower relative risks and lower significance levels [12]. Chen et al. proposed pathway-based analysis for GWAS using supervised principal components [13]. SIR is a dimension reduction method for regression problems. However, performances of these methods have not been systematically conducted.

In this article, we compare the performances of PCA, KPCA, SPCA and SIR using simulated datasets. The remainder of this article is organized as follows. In the next section, we briefly introduce the procedure of the four SNP-set analysis methods and how to generate simulated SNP sets. Then we present simulation results comparing the type I error rate and test power of these four methods. Finally, we will apply these methods to SNP sets extracted from a real Lung Cancer GWAS data. And we will conclude with a brief discussion.

## Methods

### Ethics Statement

This collaborative study was approved by the institutional review boards of China Medical University, Tongji Medical College, Fudan University, Nanjing Medical University and Guangzhou Medical College with written informed consent from the Nanjing participants.

### PCA

Assume we have a SNP set including *p* SNPs from *n* individuals. For the *i*th individual, let 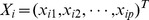 denote his/her genotypes, and the disease outcome is denoted by *Y* (1 = affected, 0 = unaffected).

PCA is a classic dimension reduction approach, which has been widely applied in genetic analysis, both for reduction of redundant information and interpretation of multiple SNPs [14]. Its basic idea is to transform *p* original variables to a new set of *k* predictor variables, which are linear combination of original variables. We use 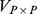 to denote the variance-covariance matrix of the SNP set. 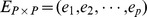 denotes the eigenvectors of 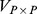, and 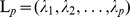 is the corresponding eigenvalues, in which 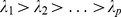. The principal components are defined by
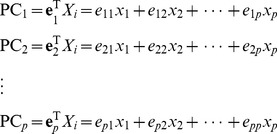



We can use PCs instead of SNPs to test the association with the disease outcome. Considering that SNPs in the SNP set are always highly correlation, the first few eigenvalues will be much greater than the others, which makes it possible to use the first few PCs to capture most of the information in the SNP set. So the first *k* PCs are needed to select for the analysis with cumulative contribution 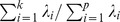 greater than some threshold. In our analysis the threshold is set as 80%. Instead of using the *p* SNPs, the first *k* PCs are used in the multiple logistic model.

### Supervised PCA

The SPCA model has been discussed in detail in Bair and Tibashirani [15], Bair et al. [16], and Chen et al. [17]. Only those SNPs with the strongest estimated correlation with the outcome are used to perform principal component analysis. The following supervised PCA model is used
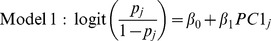
where 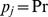 (Patient *j* has disease phenotype | PC1), and PC1 is the first principal component score estimated from the selected subset of relevant SNPs [13].

After the SNP selection step in the SPCA model, a *t*-distribution can no longer be approximated well for the test statistic, so we derive the asymptotic distribution of this statistic [13].

### Kernel PCA

Linear PCA will not always be appropriate for detect all structure in a given genomic data set. If the datasets are concentrated in a nonlinear subspace, PCA will not be suitable for detecting it. Thus, one may consider kernel principal component analysis. The kernel PCA is a nonlinear version of PCA, which is the most widely adopted among the modified PCA methods [18].

To perform KPCA, firstly, one can map the dataset x from the original space *F*_0_ into a higher-dimensional feature space *F*_1_, which is a nonlinear transform x → Φ(x), where Φ is a nonlinear function [18]. Then, we use the inner products of new feature vectors to form a kernel matrix *K*. Finally, the standard PCA is performed on the centralized kernel matrix *K*, which is the estimate of the covariance matrix of the new feature vector in *F*_1_ [12]. Such a linear PCA on *K* may be treated as a nonlinear PCA on the original data.

The nonlinear transform is based on the kernel functions, and the most common kernel functions include linear kernel, weighted linear kernel [19], radial basis function (RBF) kernel, identical-by-state (IBS) kernel and weighted IBS kernel [20]. Due to the flexibility of the RBF kernel in choosing the associated parameter, we choose the RBF kernel in present study.

### SIR

SIR is based on the inverse regression function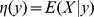 [21]. SIR can be implemented as follows.

Assume there is a dataset 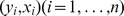. Firstly, we make use of an affine transformation to standardize the predictor x to get 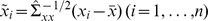, where 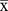 and 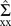 are the sample mean and covariance matrix of x respectively. Secondly, the response variable Y is sliced into H intervals,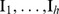, and let the proportion of the
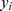
which falls in slice *h* is denoted as 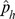, which is 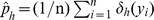.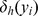 takes the values 0 or 1 depending on whether
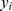
falls into the *h*th slice or not. Thirdly, we compute the sample mean of the 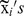 within each slice, denoted by 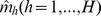, so 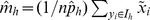. Then a (weighted) principal component analysis for 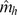 is conducted by forming the weighted covariance matrix 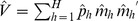, then find the eigenvalues and the eigenvectors of 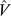. Finally, extract the *K* largest eigenvectors (row vectors)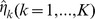 and output 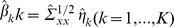.

### Simulations

To evaluate performances of PCA, SPCA, KPCA and SIR, we apply simulated datasets to measure the empirical type I error and test power. We vary the total number of the causal SNPs. So the disease model is. 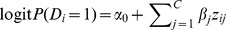



*C* is the number of causal SNPs, and in our simulations, we let *C* = 0, 1, 2 to denote null model, single causal SNP model or two causal SNPs model.

The virtual simulated datasets are generated based on the Hapsim [22], which simulates vectors from a multivariate normal distribution using a correlation matrix estimated from the MAF and joint probabilities of the original set of markers. And the Hapsim assigns a 0 or 1 for each variable along the vector using a cutoff defined by the MAF estimated from the original sample. We have improved the Hapsim program to fulfill a role to assign linkage disequilibrium (LD) structures and minor allele frequency (MAF). As it is reported that Hapsim may generate haplotypes with lower LD than the original haplotypes [23], HAPGEN2 is used for the simulations on the basis of real genes [19], [23].

### Simulations based on virtual datasets

Datasets are simulated based on virtual structures whose LD and MAF of SNPs are set artificially under the null hypothesis (H_0_) and alternative hypothesis (H_1_). We set two kinds of SNP sets, one of which has 10 SNPs and the other has 100 SNPs. Nine datasets of each SNP set are generated. Parameters of simulations are described by **Table 1**. Scenarios are set in three different MAFs (MAF = 0.05, 0.1 or 0.2 for all SNPs) and three LD structures (R^2^ = 0.2 for any two SNPs, R^2^ = 0.5 for any two SNPs, R^2^ = 0.8 for any two SNPs).

**Table 1:** Parameter settings of virtual datasets.

Scenario	MAF	LD	RR
A1	0.05	0.2/0.5/0.8	1.0
A2	0.1	0.2/0.5/0.8	1.0
A3	0.2	0.2/0.5/0.8	1.0
A4	0.05	0.2/0.5/0.8	1.3
A5	0.1	0.2/0.5/0.8	1.3
A6	0.2	0.2/0.5/0.8	1.3
A7	0.05	0.2/0.5/0.8	1.2;1.2
A8	0.1	0.2/0.5/0.8	1.2;1.2
A9	0.2	0.2/0.5/0.8	1.2;1.2

Scenarios A1–A3 are set to test performances of four methods on controlling the type I error. 1,000 cases and 1,000 controls are generated under the null disease model (C = 0), in which the outcome is independent of the loci. And 2,000 simulated datasets are produced to calculate the type I error rate. Scenarios A4–A6 are generated to compare the powers when there is only one causal SNP. Any SNP in the SNP set has the opportunity to be the causal SNP. The genetic relative risk (GRR) of these three scenarios is set 1.3. We also set two causal SNPs in scenarios A7–A9 to compare powers. GRRs of two causal SNPs are both 1.2. Parameter setting of scenarios A4–A9 is similar to scenarios A1–A3. For scenarios A4–A9, 1,000 datasets are simulated, respectively, and each dataset contains 1,000 cases and 1,000 controls. The test power is computed as the proportion of *p*-values less than 0.05.

### Simulations based on the *CLPTM1L* gene

We select the cleft lip and palate transmembrane protein 1-like (*CLPTM1L*) gene region to generate the simulated data. This gene is located at Chr 5: 1371007...1398002. The phased haplotype downloaded from the International HapMap Project (Phase 2, release 24) includes 29 SNPs within the range of 20 kb upstream and downstream. Rs31489 and rs401681 in this gene have been reported to be associated with non-small cell lung cancer (NSCLC) [24]–[26]. Simulation is based on the CEU (CEPH [Utah residents with ancestry from northern and western Europe]) population.

We generate 11 scenarios (scenarios B1–B11) of simulations, as shown by **Table 2**. We simulate scenario B1 to evaluate the performances on controlling type I error. 1000 cases and 1000 controls are generated under the null disease model (*C* = 0). Under H_0_, 2000 replicates are conducted at three significance levels (0.05, 0.01 and 0.001). Scenario B2 is the single causal SNP model to compare test powers. Each of the 29 SNPs is defined as the causal SNP in turn. The GRR is set as 1.2. Under H_1_, we repeat 1000 simulations at the significance level of 0.05. In order to make the simulations more realistic, we just use 8 of the 29 SNPs directly genotyped by the Illumina 610 k Quad chip.

**Table 2:** Parameter settings based on the *CLPTM1L* gene.

Feature of the		Number of	Locations of	Designed
simulated SNP set	Scenario	causal SNPs	the causal SNPs	RR
	B1	0	-	1.0
	B2	1	1 of 29 in turn	1.2
	B3	2	5 and 14	1.1
	B4	2	13 and 14	1.1
	B5	2	14 and 15	1.1
CLPTM1L	B6	2	15 and 28	1.1
(29 SNPs)	B7	2	6 and 13	1.1
5p13.33	B8	2	15 and 16	1.1
	B9	2	6 and 26	1.1
	B10	3	5, 14and 15	1.1
		3	5, 15 and 16	1.1
		3	5, 15 and 28	1.1
	B11	4	5, 14, 15 and 28	1.1
		4	5, 14, 15 and 16	1.1
		4	14, 15, 16 and 28	1.1

We also examine the ability of these methods on utilizing information from multiple loci assuming that there are 2 or more causal SNPs with *GRR* = 1.1 [19]. In scenario B3–B4, both of the two causal SNPs are genotyped. Only one of the two causal SNPs is genotyped in scenarios B5–B7and no causal SNPs are genotyped in scenario B8 and B9. Besides the number of genotyped causal SNPs, other differences among scenarios B3–B9 include the different median R^2^ between the causal SNPs and the genotyped SNPs and MAF of these SNPs. Details of these scenarios are shown in the first 6 columns of **Table 3**. In scenario B10 and B11, we assume that there are 3 and 4 causal SNPs, respectively. For each of the scenarios, 1000 datasets are simulated.

**Table 3:** Test power at the significance level of 0.05 for four methods in Scenarios B3–B9.

					Median *R* ^2^ with the				
Scenario	The causal SNPs	Genotyped	MAF	Position	genotyped SNPs	PCA	SPCA	KPCA	SIR
B3	rs4975616	Yes	0.417	5	0.142	0.606	0.914	0.707	0.463
	rs401681	Yes	0.433	14	0.205				
B4	rs10073340	Yes	0.133	13	0.202	0.259	0.502	0.314	0.182
	rs401681	Yes	0.433	14	0.205				
B5	rs401681	Yes	0.433	14	0.205	0.590	0.882	0.708	0.483
	rs466502	No	0.425	15	0.207				
B6	rs466502	No	0.425	15	0.207	0.588	0.894	0.635	0.420
	rs27061	Yes	0.442	28	0.207				
B7	rs6554759	No	0.144	6	0.178	0.276	0.208	0.236	0.201
	rs10073340	Yes	0.133	13	0.202				
B8	rs466502	No	0.425	15	0.207	0.598	0.881	0.693	0.478
	rs465498	No	0.433	16	0.210				
B9	rs6554759	No	0.144	6	0.178	0.145	0.129	0.136	0.161
	rs27064	No	0.142	26	0.224				

### Simulations based on the *XRCC1* gene

The X-ray repair cross-complementing protein 1 (*XRCC1*), which has 24 SNPs in 19q13.2, is a key DNA repaired gene. Polymorphism of *XRCC1* may increase cancer risk and significantly alter patient responses to chemotherapy [27], [28]. The reference haplotype is downloaded from the HapMap (Phase 2, release 24) based on the CEU population. We conduct four scenarios of simulations based on the region (scenarios C1–C4). Parameters of the simulations are described by **Table 4**. Scenario C1 is generated to evaluate the type I error while C2 to C4 evaluate the test powers. 2000 simulated replicates are with no association between the disease outcome and the SNPs in scenario C1. In scenario C2, each of the 24 SNPs is set to be the causal SNP with a *GRR* of 1.2 in turn. Again, although the simulated datasets are generated using the overall 24 SNPs, only 5 genotyped SNPs are used in the analyses. Scenarios C3 and C4 have two causal SNPs with *GRR* = 1.1. For scenarios C2–C4, 1000 simulated datasets are generated to calculate test powers, and each dataset contains 1,000 cases and 1,000 controls.

**Table 4:** Parameter settings based on the *XRCC1* gene.

Feature of the		Number of	Locations of	Designed
simulated SNP set	Scenario	causal SNPs	the causal SNPs	RR
	C1	0	-	1.0
XRCC1(24 SNPs)	C2	1	1 of 24 in turn	1.2
19q13.2	C3	2	4 and 24	1.1
	C4	2	9 and 19	1.1

### Application of PCA, SPCA, KPCA and SIR to a real GWAS dataset

These methods are applied to a real GWAS dataset studying the genetic susceptibility of NSCLC. Details of the study have been described previously [26]. A large-scale GWAS of lung cancer in Han Chinese populations was performed by genotyping SNPs using Affymetrix Genome-wide Human SNP Array 6.0 chips. In this study, only 1,473 cases, 1,962 controls and 570,373 SNPs are analyzed. To illustrate the performance of these methods, we mainly focus on 2 regions from the dataset. The first one includes 8 SNPs in *CLPTM1L* gene which is associated with nicotine addiction, smoking behavior and NSCLC [29]–[31]. The length of the second region is about 208.4 kb, which includes 15 SNPs in 6p21.32–21.33. Genes in this region include the *TNXB*, *FKBPL* and *PPT2*.Gene expression of *TNXB* was reported to be association with lung squamous cell cancer [32] and *PPT2* was association with pulmonary function [33]. *FKBPL* has been proposed as a novel prognostic and predictive biomarker [34].

Analyses of the simulated datasets and actual datasets are performed using R package (version 2.13). The “*superpc*”, “*kernlab*”, and “*dr*” packages are used to conduct SPCA, KPCA and SIR analyses, respectively.

## Results

### Results based on virtual datasets

#### Empirical type I error rate

The empirical type I error rates of PCA, SPCA, KPCA and SIR based on 10 SNPs are presented by **Table 5**, **Table S1**–**S2 in File S1**. All of these methods can control the type I error at the significance level of 0.05, 0.01or 0.001. For 100 SNPs in a dataset, PCA, SPCA and KPCA control the type I error at the significance levels in most situations by **Table 6**, **Table S3**–**S4 in File S1**, while SIR controls the type I error strictly.

**Table 5:** Empirical type I error rates at the significance level of 0.05 based on 10 SNPs.

MAF	LD	PCA	SPCA	KPCA	SIR
0.05	0.2	0.054	0.049	0.053	0.050
	0.5	0.062	0.047	0.058	0.052
	0.8	0.056	0.059	0.057	0.046
0.1	0.2	0.041	0.047	0.041	0.039
	0.5	0.046	0.050	0.043	0.043
	0.8	0.052	0.046	0.047	0.044
0.2	0.2	0.048	0.051	0.045	0.048
	0.5	0.045	0.049	0.050	0.044
	0.8	0.056	0.046	0.048	0.051

**Table 6:** Empirical type I error rates at the significance level of 0.05 based on 100 SNPs.

MAF	LD	PCA	SPCA	KPCA	SIR
0.05	0.2	0.050	0.049	0.055	0.040
	0.5	0.046	0.046	0.054	0.026
	0.8	0.017	0.042	0.045	0.002
0.1	0.2	0.063	0.048	0.061	0.051
	0.5	0.058	0.055	0.058	0.035
	0.8	0.031	0.048	0.049	0.011
0.2	0.2	0.069	0.050	0.071	0.043
	0.5	0.059	0.047	0.061	0.038
	0.8	0.049	0.046	0.050	0.037

#### Empirical test power with single causal SNP

**Figure 1:**
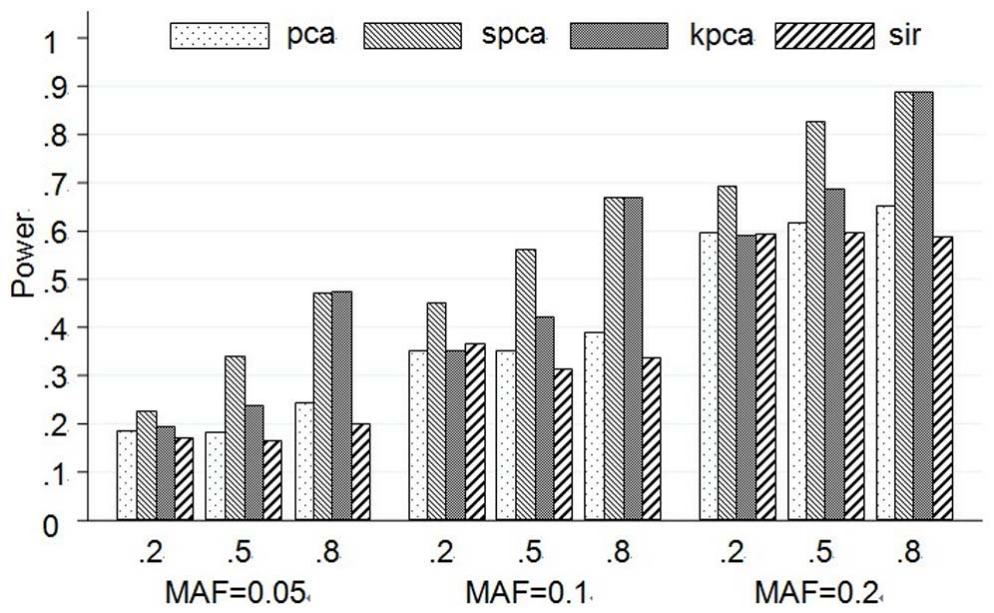
Test powers at single causal SNP model based on 10 SNPs. The plot shows the powers (y-axis) of each method over the different LD and MAF structures (x-axis). The first line of x-axis represents LD, and the bottom line is MAF.

Results from the simulations on scenarios A4–A6 are presented by **Figure 1**, which shows that SPCA has the best power. As MAF is fixed as 0.05, 0.1 or 0.2 and LD is set as 0.2, powers of PCA,KPCA and SIR are approximate, which are respectively near 20%, 35% and 60%. At the same time, the power of SPCA is 22.6%, 45.1% and 69.2%. When LD is 0.5, KPCA is about 7% more powerful than PCA. When LD is strong, the power of KPCA is close to that of SPCA. The power of SIR is lower than the other methods in most scenarios.

#### Empirical test power with two causal SNPs

**Figure 2:**
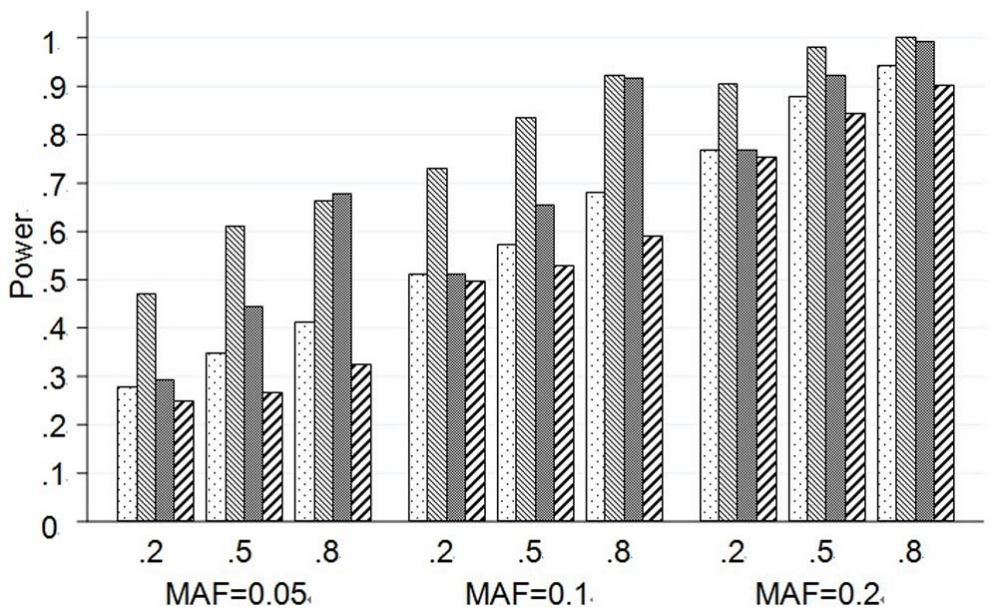
Test powers at two causal SNPs model based on 10 SNPs. The plot shows the powers (y-axis) of each method over the different LD and MAF structures (x-axis). The first line of x-axis represents LD, and the bottom line is MAF.

Results from the simulation on scenarios A7–A9 are presented by **Figure 2**. As those scenarios with two causal SNPs, the change trends of powers are nearly the same as the single causal SNP model. While the power of every scenario based on two causal SNPs is obviously higher than the single causal SNP model. And when MAF is set as 0.2, no matter what the LD structure is, the powers of four methods are close to or greater than 80%.

The change trends of test power of 100 SNPs are similar with those of 10 SNPs, and the detailed results are listed in **Figure S1 and S2 in File S1**. Powers of PCA and KPCA are much lower than SPCA in most situations.

### Results based on the *CLPTM1L* gene

#### Empirical type I error rate

PCA, SPCA, KPCA and SIR can control the empirical type I error at the significance level of 0.05, 0.01 or 0.001. Results are presented by **Table 7**.

**Table 7:** Empirical type I error based on the real genes.

Gene	Scenario	α	PCA	SPCA	KPCA	SIR
CLPTM1L	B1	0.05	0.040	0.050	0.045	0.048
		0.01	0.008	0.009	0.008	0.008
		0.001	0.001	0.001	0.000	0.001
XRCC1	C1	0.05	0.056	0.046	0.050	0.059
		0.01	0.012	0.008	0.012	0.012
		0.001	0.001	0.001	0.000	0.001

#### Empirical test power with single causal SNP

**Figure 3:**
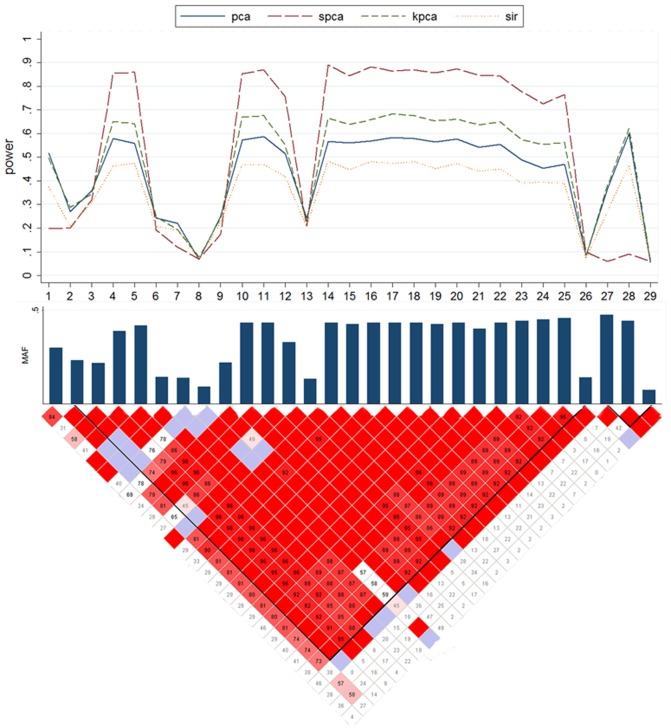
Powers of the causal SNP in Scenario B2 based on the *CLPTM1L* gene. The top plot shows the power (y-axis) of each method over the locations (x-axis) of the causal SNPs. The bar-plot shows the MAFs of all SNPs. The LD structure of the 29 SNPs is shown by the heat plot in the bottom of the figure, in which the red scale indicates the value of R^2^ (1 = red, 0 = white).

Results of scenario B2 are presented by **Figure 3**. On the basis of Figure 3, we can examine how test powers of each method vary with MAF and LD of the causal SNP. In general, all methods have power when the causal SNP is in high LD with the other SNPs. In most occasions, SPCA still has the greatest power, which is followed by KPCA. When the MAF of the causal SNP is low, powers of four methods are all weak, which are only about 10%. It is worth noticing that though PCA does not have good performance in general, it has greater power than the other under this situation. For example, the causal SNP is at one of the 6th–7th and 13th loci.

#### Empirical test power with more than one causal SNP

**Table 3** shows the powers of scenarios B3 to B9. Once again, SPCA has the greatest power in most situations, while SIR is the worst one. We can see that the powers of the four methods are almost independent of the LD between the causal and genotyped SNPs. However, the test powers are decreased by the low MAF of the causal SNPs. Although powers of all methods are weak, it is also interesting to find that PCA is slightly superior to the other methods in scenarios B7and B9 when MAFs of the causal SNPs are low. Scenarios B10 and B11 in which there are 3 and 4 causal SNPs in the *CLPTM1L* gene yield similar conclusion that tests combining multiple SNPs tend to have higher power (**Table S5** **in File S1**).

### Results based on the *XRCC1* gene

#### Empirical type I error rate

PCA, SPCA, KPCA and SIR can control the empirical type I error at the significance level of 0.05, 0.01 or 0.001. Results are presented by **Table 7**.

#### Empirical test powers

**Figure 4:**
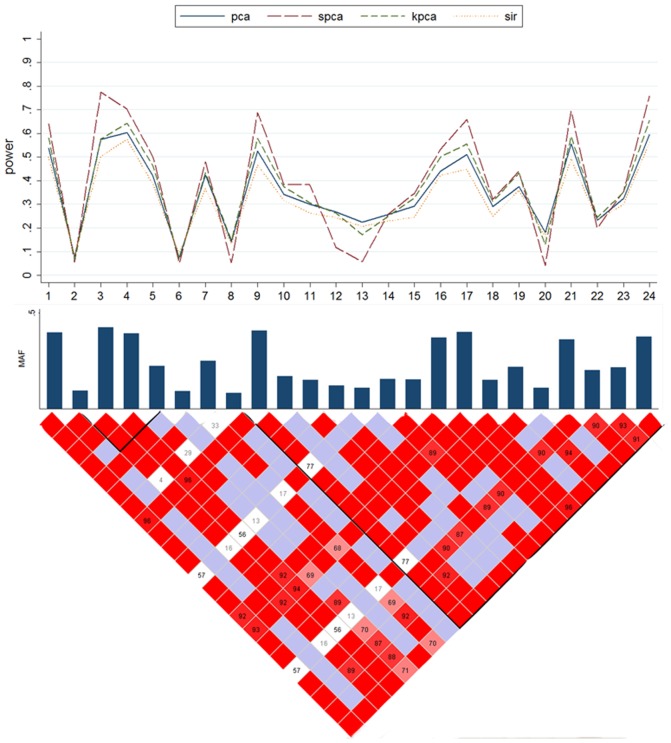
Powers of the causal SNP in Scenario C2 based on the *XRCC1* gene. The top plot shows the power (y-axis) of each method over the locations (x-axis) of the causal SNPs. The bar-plot shows the MAFs of all SNPs. The LD structure of the 24 SNPs is shown by the heat plot in the bottom of the figure, in which the red scale indicates the value of R^2^ (1 = red, 0 = white).

Results of scenario C2 are shown by **Figure 4**, by which we also can evaluate how test powers of each method vary with MAF and LD of the causal SNPs. The change trends of test power are similar with those of the *CLPTM1L* gene. All of the four methods have statistical power when the causal SNP is in strong LD with the other SNPs. Some SNPs do not have high MAF and LD structures, so four methods have low powers, such as the 2th, 6th, and 7th loci. Again, SPCA has the best power in most situations. We also find that SPCA has much better performances than the other methods especially when the causal SNPs have high MAF. Simulations from scenarios C3–C4 generate similar results (**Table S6 in File S1**) and they also show that tests combining multiple SNPs tend to have higher power.

### Run time

As shown in **Table S7 in File S1**, PCA and SIR both take around 2 minute for each simulation, faster than SPCA for each simulation. KPCA will likely require hours while the other methods will likely require minutes. So KPCA needs more computational-resources consuming.

### Applications on Lung Cancer GWAS

The results of the analysis are shown in **Table 8**. For the SNP set from the *CLPTM1L* gene, rs465498 yields the least *p*-value of 2.19E-4 (1.75E-3 after the Bonferroni correction for the effective number of tests). The *p*-value of SPCA is 9.00E-4, the least of four methods. We extract the 4 and 3 PCs for PCA and KPCA to calculate *p*-values, respectively. *P*-values of KPCA, PCA and SIR are 5.55E-3,1.25E-2 and 3.08E-2, respectively. For SNP set 2, the result also shows that SPCA performs the best, and its *p*-value is 4.00E-4. Rs3130284 yields the least *p*-value of 5.01E-4 (7.51E-3 after the Bonferroni correction for the effective number of tests). Meanwhile, for the SNP sets, we regress the disease outcome on the first PC of PCA and KPCA. We find that PCA has better performance, and even superior to KPCA when only the 1st PC is analyzed.

**Table 8:** Results of four methods on the analysis of the SNP set from the GWAS study.

	Individual SNP analysis							
	The least *p*-value	*p*-value for						
SNP set	in the SNP set	the SNP set	PCA	SPCA	KPCA	SIR	PCA(1)^a^	KPCA(1)^a^
1	2.19E-4	1.75E-3	1.25E-2	9.00E-4	5.55E-3	3.08E-2	1.98E-3	1.01E-2
2	5.01E-4	7.51E-3	7.18E-2	4.00E-4	2.71E-2	2.28E-3	1.86E-3	8.07E-1

a We extract the 1st PC to analyze.

**Figure 5:**
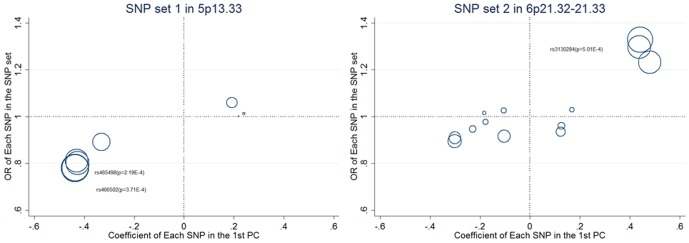
Loadings of the significant PCs on each of the SNP sets from 5p13.33 and 6p21.32–21.33. The diameters of the circles in the plots are proportional to −log10(*p*-value).

To understand how PCA utilizes the information from multiple SNPs, we also examine the coefficient of each SNP in the top PCs. For the SNP sets, we regress the phenotype on the top PCs. The 1st PC from the two SNP sets, which is significant, is then showed by **Figure 5**. The significant PCs tend to have heavy loading on the “important” SNPs. As an example, for SNP set 1, the 1st PC has heavy loadings on rs465498 (odds ratio OR = 0.78, *p*-value = 2.19E-4) and rs466502 (OR = 0.79, *p*-value = 3.71E-4). Rs465498 in the *CLPTM1L* gene has been reported to be associated with non-small cell lung cancer (NSCLC) [26].

## Discussion

In genome-wide association studies, SNP set association analyses are applied in order to reduce the number of hypothesis tests analysis and is also one of the dimension reduction methods. On the basis of the SNP set association analysis, we can screen potential SNPs association with the disease and do further analysis. To reduce the false positive rate caused by multiple testing, some researchers have proposed PCA-based methods and have found that these methods are more powerful than single locus test and haplotype-based test [11], [35]–[37].

In our research, we compare the performances of PCA, SPCA, KPCA and SIR on testing the association for the analysis of GWAS. We conduct extensive simulated datasets based on the virtual structures and the haplotypes downloaded from the International HapMap Project, and also apply these methods to two SNP sets extracted from the GWAS data on NSCLC. The results suggest that four methods can control the type I error and have the ability to test the association between the outcome and the SNP set. If the causal SNP(s) has/have strong LD or high MAF, these methods can combine the information and provide better test power. In addition, if there are two or more causal SNPs in a SNP set, methods can combine their information and provide higher test power than one causal SNP.

Many complex diseases are influenced by joint effects of genetic variations in multiple SNPs. In this paper, we have outlined some strategies for conducting SNP set analysis for GWAS data. In our analysis, results show that PCA-based methods are better than SIR. We have studied how PCA utilizes the information from multiple SNPs in NSCLC analysis. The analysis suggests that the significant PCs tend to have heavy loading on the important SNPs, indicating that the results obtained from PCA-based methods may be “biologically” easy to be explained. PCA is an effective method for testing association of joint effects of genetic variations in multiple SNPs. However, one weakness of PCA is that the latent variables identified by the PCs may or may not be related to the outcome [13]. Thus, without a SNP screening step, using all SNPs to summarize information can result in reducing test power for SNP set-based analysis, due to the inclusion of SNPs unrelated to the disease.

SPCA is a supervised dimension reduction method, which removes some irrelevant SNPs before extracting principal components. Thus, in lung cancer GWAS analysis, when we use the 1st PC to analyze in PCA and SPCA, results show that *p-*values of SPCA are smaller than those of PCA. And simulated results also show that this approach performs better than PCA in most situations. A possible limitation of SPCA is that we use only the 1st PC in the analysis, which takes the risk of missing the causal SNP(s). As an example, in scenario C2, when SNPs have low MAF, powers of SPCA decrease dramatically, and are even inferior to other methods in some scenarios. The 1st PC may fail to catch the information from the causal SNPs with low MAF. To improve the power of SPCA, a possible solution is to exclude the PCs independent of the causal SNPs.

Gao et al. [12] showed that KPCA performed well under null hypothesis, and powers of KPCA were higher than PCA under different situations. In our research, we also demonstrate that KPCA has better power than PCA in most occasions. Owing to KPCA doesn't screen the irrelevant SNPs, powers of KPCA may be inferior to those of SPCA. And we find that KPCA needs more computational-resources.

After standardizing x, SIR conducts with a crude estimate of the inverse regression curve E(X | y), which is the slice mean of x after slicing the range of y into several intervals and partitioning the whole data into several slices according to the y value. A principal component analysis is then applied to these slice means of x. However, our results show that the powers of SIR are almost the lowest throughout the simulations. One possible reason is that the phenotype is a binary variable and can be divided into only two slices, leading to low statistical powers. Thus, SIR is not recommended to apply in GWAS when the phenotype is of two classes.

Test powers from PCA and KPCA are affected by the number of principal components extracted in the analysis. As an example, in the NSCLC analysis, when we extract the 1st PC to do regression in the SNP set 1, *p*-value of PCA (*p* = 1.98E-3) is much smaller than that using PCs explaining at least 80% of total variation, and may have even better performance than KPCA. Some studies suggest that when the SNP set is with simple LD structure, only a very small number of PCs are needed to explain a large proportion (40%) of the total variation, and results from PCA (20%) are the same as those from 40% [19]. If only the 1st PC is needed to capture the information of the causal SNP, including more PCs will decrease the power. This is also demonstrated by the fact that when we all select PCs with cumulative contribution greater than 80% in our analysis, powers of PCA and KPCA are very low in most situations if the SNP set contains 100 SNPs. More degrees of freedom are exhausted due to that we have to include a lot of redundant PCs. However, just like KPCA, using only the 1st PC, regardless of whether the 1st PC catches the information from causal SNPs, may also lead to decreased power. Thus, it is critical to examine the LD structure and MAF of the SNP set before determining the analysis method. So choosing the appropriate number of PCs in PCA and KPCA may be very important and not be an easy task. LD structure and MAF should be carefully examined [19].

Another advantage of PCA-based methods is that the result of PCA is easy to be explained. Firstly, a significant SNP set in PCA-based methods can be followed by a fine mapping or deep sequencing to identify the true causal SNP, which should reside in the region in or close to the significant SNP set. Secondly, by checking the loading of the significant PCs on each SNP, we can find which SNPs are more associated with the disease. Thirdly, the principal component score can also be used as a risk score for the risk of developing the disease.

There are several limitations in our study. First, we just use PCA and KPCA with 80% PCs to analyze SNP sets. Effect of the number of PCs using by PCA-based methods is not thoroughly examined. Second, more complicated situations, such as rare variations and gene-gene interaction, are not included in this article. Further work to solve such problems will certainly be warranted.

## Supporting Information

File S1 Figure S1, Test powers at single causal SNP model based on 100 SNPs. The plot shows the powers (y-axis) of each method over the different LD and MAF structures (x-axis). The first line of x-axis represents LD, and the bottom line is MAF. Figure S2, Test powers at two causal SNPs model based on 100 SNPs. The plot shows the powers (y-axis) of each method over the different LD and MAF structures (x-axis). The first line of x-axis represents LD, and the bottom line is MAF. Table S1, Empirical type I error rates at the significance level of 0.01based on 10 SNPs. Table S2, Empirical type I error rates at the significance level of 0.001 based on 10 SNPs. Table S3, Empirical type I error rates at the significance level of 0.01based on 100 SNPs. Table S4, Empirical type I error rates at the significance level of 0.001 based on 100 SNPs. Table S5, Test power at the significance level of 0.05 for four methods in Scenarios B10-B11. Table S6, Test power at the significance level of 0.05 for four methods in Scenarios C3–C4. Table S7, Run time comparison.
(DOCX)
