# Supplementary material for: SNP Set Association Analysis for Genome-Wide Association Studies
Source: PLoS One. 2013 May 3;8(5):e62495. doi: 10.1371/journal.pone.0062495 (PMC3643925; doi:10.1371/journal.pone.0062495)
Supplement: File S1 — Figure S1, Test powers at single causal SNP model based on 100 SNPs. The plot shows the powers (y-axis) of each method over the different LD and MAF structures (x-axis). The first line of x-axis represents LD, and the bottom line is MAF. Figure S2, Test powers at two causal SNPs model based on 100 SNPs. The plot shows the powers (y-axis) of each method over the different LD and MAF structures (x-axis). The first line of x-axis represents LD, and the bottom line is MAF. Table S1, Empirical type I error rates at the significance level of 0.01based on 10 SNPs. Table S2, Empirical type I error rates at the significance level of 0.001 based on 10 SNPs. Table S3, Empirical type I error rates at the significance level of 0.01based on 100 SNPs. Table S4, Empirical type I error rates at the significance level of 0.001 based on 100 SNPs. Table S5, Test power at the significance level of 0.05 for four methods in Scenarios B10-B11. Table S6, Test power at the significance level of 0.05 for four methods in Scenarios C3–C4. Table S7, Run time comparison. (DOCX) [file pone.0062495.s001.docx]

**Table S1. Empirical type I error rates at the significance level of 0.01based on 10 SNPs**

| MAF | LD | PCA | SPCA | KPCA | SIR |
| --- | --- | --- | --- | --- | --- |
| 0.05 | 0.2 | 0.009 | 0.010 | 0.010 | 0.010 |
|  | 0.5 | 0.016 | 0.010 | 0.010 | 0.010 |
|  | 0.8 | 0.011 | 0.011 | 0.011 | 0.006 |
| 0.1 | 0.2 | 0.007 | 0.009 | 0.006 | 0.010 |
|  | 0.5 | 0.001 | 0.008 | 0.006 | 0.009 |
|  | 0.8 | 0.013 | 0.009 | 0.010 | 0.009 |
| 0.2 | 0.2 | 0.010 | 0.010 | 0.009 | 0.008 |
|  | 0.5 | 0.011 | 0.012 | 0.009 | 0.007 |
|  | 0.8 | 0.011 | 0.009 | 0.009 | 0.009 |

**Table S2. Empirical type I error rates at the significance level of 0.001 based on 10 SNPs**

| MAF | LD | PCA | SPCA | KPCA | SIR |
| --- | --- | --- | --- | --- | --- |
| 0.05 | 0.2 | 0.001 | 0.001 | 0.001 | 0.000 |
|  | 0.5 | 0.001 | 0.001 | 0.001 | 0.001 |
|  | 0.8 | 0.001 | 0.000 | 0.001 | 0.001 |
| 0.1 | 0.2 | 0.001 | 0.001 | 0.001 | 0.001 |
|  | 0.5 | 0.000 | 0.000 | 0.000 | 0.000 |
|  | 0.8 | 0.001 | 0.001 | 0.001 | 0.000 |
| 0.2 | 0.2 | 0.001 | 0.001 | 0.001 | 0.001 |
|  | 0.5 | 0.000 | 0.002 | 0.001 | 0.000 |
|  | 0.8 | 0.001 | 0.000 | 0.001 | 0.001 |

**Table S3. Empirical type I error rates at the significance level of 0.01based on 100 SNPs**

| MAF | LD | PCA | SPCA | KPCA | SIR |
| --- | --- | --- | --- | --- | --- |
| 0.05 | 0.2 | 0.010 | 0.009 | 0.024 | 0.009 |
|  | 0.5 | 0.006 | 0.008 | 0.015 | 0.003 |
|  | 0.8 | 0.002 | 0.009 | 0.010 | 0.000 |
| 0.1 | 0.2 | 0.014 | 0.007 | 0.021 | 0.009 |
|  | 0.5 | 0.010 | 0.008 | 0.010 | 0.004 |
|  | 0.8 | 0.003 | 0.011 | 0.010 | 0.001 |
| 0.2 | 0.2 | 0.014 | 0.010 | 0.019 | 0.005 |
|  | 0.5 | 0.010 | 0.010 | 0.010 | 0.008 |
|  | 0.8 | 0.010 | 0.011 | 0.013 | 0.003 |

**Table S4. Empirical type I error rates at the significance level of 0.001 based on 100 SNPs**

| MAF | LD | PCA | SPCA | KPCA | SIR |
| --- | --- | --- | --- | --- | --- |
| 0.05 | 0.2 | 0.000 | 0.001 | 0.000 | 0.001 |
|  | 0.5 | 0.001 | 0.001 | 0.001 | 0.000 |
|  | 0.8 | 0.000 | 0.001 | 0.001 | 0.000 |
| 0.1 | 0.2 | 0.002 | 0.001 | 0.001 | 0.001 |
|  | 0.5 | 0.001 | 0.000 | 0.002 | 0.001 |
|  | 0.8 | 0.000 | 0.002 | 0.002 | 0.000 |
| 0.2 | 0.2 | 0.002 | 0.001 | 0.002 | 0.001 |
|  | 0.5 | 0.001 | 0.002 | 0.000 | 0.001 |
|  | 0.8 | 0.001 | 0.001 | 0.001 | 0.000 |

**Table S5.** **Test power at the significance level of 0.05 for four methods in Scenarios B10-B11**

| Scenario | PCA | SPCA | KPCA | SIR |
| --- | --- | --- | --- | --- |
| B10 | 0.976 | 0.998 | 0.990 | 0.947 |
|  | 0.981 | 1.000 | 0.991 | 0.956 |
|  | 0.775 | 0.923 | 0.861 | 0.666 |
| B11 | 0.993 | 1.000 | 0.998 | 0.977 |
|  | 1.000 | 1.000 | 1.000 | 1.000 |
|  | 0.998 | 1.000 | 1.000 | 0.990 |

**Table S6. Test power at the significance level of 0.05 for four methods in Scenarios C3-C4**

|  |  |  |  |  | Median *R*^2^ with |  |  |  |  |
| --- | --- | --- | --- | --- | --- | --- | --- | --- | --- |
| Scenario | The causal SNPs | Genotyped | MAF | Position | the genotyped SNPs | PCA | SPCA | KPCA | SIR |
| C3 | rs1799778 | Yes | 0.392 | 4 | 0.149 | 0.501 | 0.799 | 0.566 | 0.437 |
|  | rs3213255 | Yes | 0.375 | 24 | 0.119 |  |  |  |  |
| C4 | rs1001581 | No | 0.407 | 9 | 0.111 | 0.312 | 0.544 | 0.330 | 0.262 |
|  | rs2854509 | No | 0.217 | 19 | 0.114 |  |  |  |  |

**Table S7. Run time comparison**

| Datasets | Numbers of SNP | PCA | SPCA | KPCA | SIR |
| --- | --- | --- | --- | --- | --- |
| Virtual | 10 | 2min.15sec. | 7min.27sec. | 4h.32min. | 2min.13sec. |
|  | 100 | 15min.25sec. | 34min.11sec. | 9h.20min. | 18min.44sec. |
| Actual | 29(8) | 1min.56sec. | 10min.7sec. | 4h.10min. | 2min.10sec. |
|  | 24(5) | 1min.55sec. | 7min.23sec. | 5h.48min. | 1min.50sec. |

The programs were run on a Dell server with 6 x Quad core Intel(R) Xeon(R) CPUs X5690 @3.47GHz and 32GB of RAM running CentOS release 5.3 Linux Distribution.

**
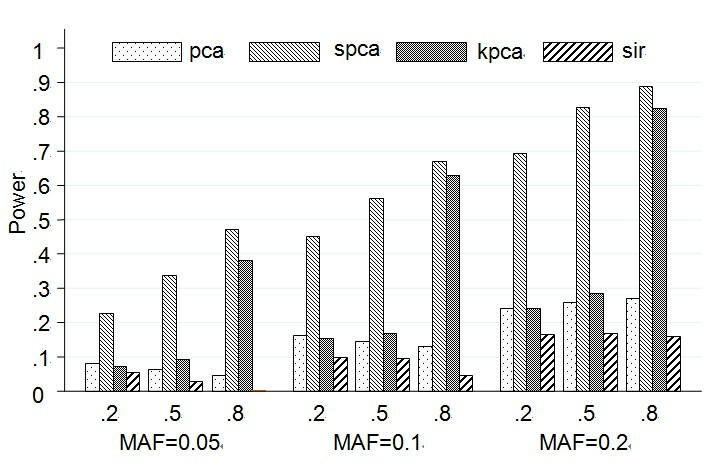
**

**Figure S1: Test powers at single causal SNP model based on 100 SNPs.** The plot shows the powers (y-axis) of each method over the different LD and MAF structures (x-axis). The first line of x-axis represents LD, and the bottom line is MAF.

**
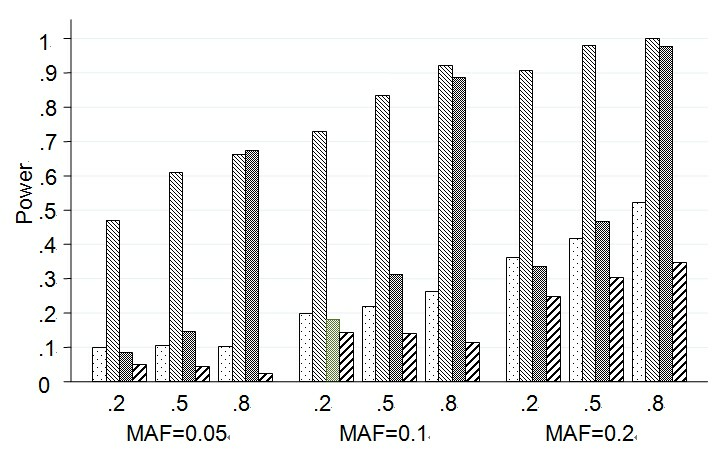
**

**Figure S2: Test powers at two causal SNPs model based on 100 SNPs.** The plot shows the powers (y-axis) of each method over the different LD and MAF structures (x-axis). The first line of x-axis represents LD, and the bottom line is MAF.
